# Positive Correlation between IP-10 and IFN-*γ* Levels in Rhesus Monkeys* (Macaca mulatta)* with Either Naturally Acquired or Experimental Infection of* Mycobacterium tuberculosis*

**DOI:** 10.1155/2017/5089752

**Published:** 2017-04-20

**Authors:** Fangui Min, Ruike Wu, Jinchun Pan, Shuwu Huang, Yinzhu Luo, Yu Zhang

**Affiliations:** Guangdong Laboratory Animals Monitoring Institute, Guangdong Provincial Key Laboratory of Laboratory Animals, Guangzhou 510663, China

## Abstract

Numerous studies identify that IP-10 and IFN-*γ* are involved in leucocyte migration and activation and regarded as promising surrogate biomarkers in human and bovine tuberculosis infection, but there is lack of evidence for IP-10 in nonhuman primates. In this study, we directly determined IP-10 and IFN-*γ* levels in plasma from 30 healthy monkeys, 30 monkeys with naturally acquired tuberculosis, 4 monkeys experimentally infected with tuberculosis, and PPD stimulated whole blood of 14 monkeys with naturally acquired tuberculosis by ELISA. Higher plasma levels of IP-10 and IFN-*γ* were observed in natural tuberculosis monkeys than in healthy controls. The dynamic changes of plasma IP-10 and IFN-*γ* in experimental infections showed consistent representation of a transient increase during the infection period. After PPD stimulation, release of IP-10 and IFN-*γ* is significantly induced in natural tuberculosis monkeys, but the stimulation index of IP-10 was significantly lower than IFN-*γ*. Further analysis showed that positive correlation between IP-10 and IFN-*γ* existed in healthy and tuberculosis monkeys. Our findings support plasma IP-10 and IFN-*γ* as biomarkers for monitoring ongoing inflammation of nonhuman primate tuberculosis, and IFN-*γ* is a more valuable diagnostic biomarker.

## 1. Introduction

IFN-*γ*-inducible protein 10 (IP-10), also named CXCL10, belongs to the CXC-chemokine family. As a chemokine, IP-10 is involved in delayed type hypersensitivity [1], which can activate monocytes, enhance Th1 responses, and elevate IFN-*γ* gene expression [2].

A number of reports suggest that IP-10 is involved in tuberculosis infection. For human tuberculosis patients, higher IP-10 plasma levels were found compared to that of healthy contacts or controls [3], and IP-10 release was significantly enhanced after stimulation with* M. tuberculosis* specific antigens, such as PPD and RD-1 gene encoded proteins and TB7.7 [4]. For bovine tuberculosis, there were different results for IP-10 serving as an alternative or ancillary biomarker for the immunodiagnosis of bovine tuberculosis. Waters et al. reported that there was no significant difference in IP-10 responses to mycobacterial antigens between the periods before and after stimulation in cattle populations. However, IP-10 responses to mycobacterial antigens did increase after experimental infection, although IP-10 levels in nonstimulated wells also increased suggesting nonspecific production of IP-10 in vivo in response to experimental* M. bovis* infection [5]. In contrast to Waters et al.'s results, Parsons et al. suggested that IP-10 might be a valuable diagnostic biomarker of* M. bovis* infection in cattle [6]. Another report showed that IP-10 is significantly elevated in whole blood stimulated with RD-1 gene encoded proteins, produced in much greater abundance than IFN-*γ* in* M. bovis*-infected buffaloes [7]. Till now, few reports relative to IP-10 were seen on monkeys.

IFN-*γ* is an important cytokine relative to many diseases, and high expression of the IFN-*γ* gene may carry a higher risk. IFN-*γ* is also one of the most important proinflammatory cytokines involved in tuberculosis infection and immunity and serves as biomarker for the immunodiagnosis of tuberculosis in human patients and animal infections, including monkeys [8–11].

The present study was designed to analyze the correlation between IP-10 and IFN-*γ* in healthy and tuberculosis monkeys and assess whether plasma levels of IP-10 might represent useful clinical tools for monitoring ongoing inflammation in monkeys with tuberculosis.

## 2. Materials and Methods

### 2.1. Plasma Samples

Thirty plasma samples of captive rhesus monkeys* (Macaca mulatta)* were confirmed to be naturally infected with tuberculosis by routine quarantines (semiannual TSTs) and further necropsies were included in this study. Serial plasma of 0, 6, 10, 12, 18, and 22 weeks from 4 rhesus monkeys experimentally infected with* M. tuberculosis* H37Rv was also used in this study. The infection model was reported previously [12], including 2 monkeys (06-1523R and 06-1519R) treated with high doses of 500 CFU bacteria and 2 monkeys (06-1445R and 06-1411R) treated with low doses of 50 CFU bacteria. Heparinized whole blood from 14 rhesus monkeys showing positive reactions for repeated tuberculin skin testing was incubated with PPD and PBS as reported [4]. The supernatant plasma was harvested for this study. Control plasma was from 30 healthy rhesus monkeys free of tuberculosis (showing negative TST reactions). All plasma were collected and stored at −80°C.

### 2.2. Determination of IP-10 in Plasma

The concentrations of IP-10 in plasma were determined by standard enzyme linked immunosorbent assay (ELISA) kits according to the manufacturer's protocol, which were from Shanghai Gaining biological Co. The concentrations were expressed as pg/ml plasma.

### 2.3. Determination of IFN-*γ* in Plasma

IFN-*γ* plasma concentrations were measured with ELISA kits from Mabtech Inc. (Ohio, USA) and expressed as pg/ml plasma.

### 2.4. Data Analysis

Results were expressed as mean ± SD. Between-group differences were analyzed by unpaired and paired *t*-test. Spearman's rank correlation coefficient was used to analyze the correlation between IP-10 and IFN-*γ* plasma concentrations. *p* values less than 0.05 were considered statistically significant.

## 3. Results

### 3.1. Higher Levels of IP-10 and IFN-*γ* in Monkeys with Naturally Acquired Tuberculosis Compared to Healthy Controls

**Figure 1:**
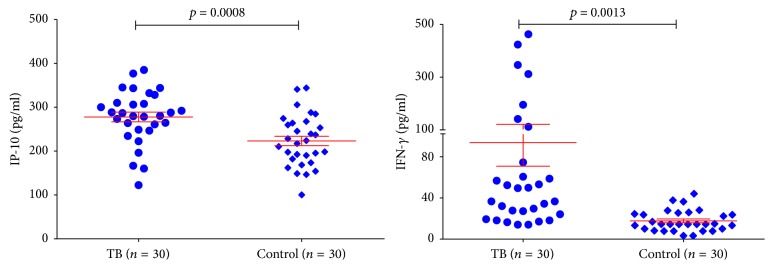
*Basic concentrations of serum IP-10 and IFN-γ in healthy and TB monkeys*. Significant differences were found between healthy and TB monkeys for both IP-10 and IFN-*γ*.

Basic concentrations of plasma IP-10 and IFN-*γ* in healthy controls (*n* = 30) and monkeys with naturally acquired tuberculosis (*n* = 30) were determined and shown in Figure 1. The average concentrations of IP-10 in healthy and tuberculosis monkeys were “225.53 ± 59.07” pg/ml and “280.77 ± 61.73” pg/ml, and concentrations of plasma IFN-*γ* in healthy and tuberculosis monkeys were “17.73 ± 10.26” pg/ml and “93.57 ± 122.77” pg/ml, respectively. Both IP-10 and IFN-*γ* plasma levels of tuberculosis monkeys were significantly higher than those of healthy monkeys (unpaired *t*-test, *p* < 0.05).

### 3.2. Positive Correlation between IP-10 and IFN-*γ* in Healthy Controls and Monkeys with Naturally Acquired Tuberculosis

**Figure 2:**
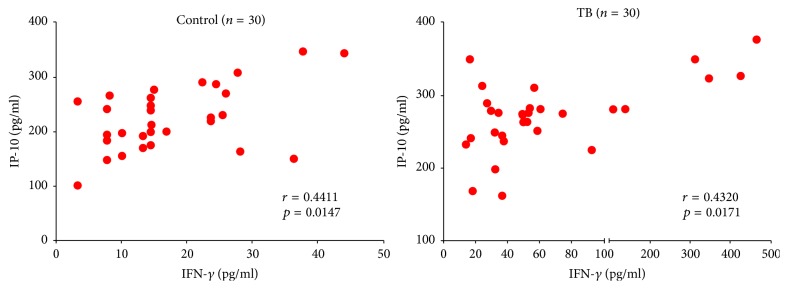
*Distribution of IP-10 and IFN-γ of healthy and natural tuberculosis*. Moderate positive correlation between IP-10 and IFN-*γ* was observed in both healthy and natural tuberculosis monkeys (0.3 < *r* < 0.5).

The determined values of healthy controls and monkeys with naturally acquired tuberculosis were used to analyze the correlation between IP-10 and IFN-*γ* by Spearman's rank correlation coefficient.  Figure 2 showed the distribution of two groups. For the healthy controls, the *r*-value was 0.4411 with a *p* value of 0.0147. And the monkeys with naturally acquired tuberculosis gave the *r*-value of 0.4320 and *p* value of 0.0171. Results showed that moderate positive correlation between IP-10 and IFN-*γ* existed in both healthy controls and monkeys with naturally acquired tuberculosis (0.3 < *r* < 0.5).

### 3.3. Positive Correlation between IP-10 and IFN-*γ* in Monkeys with Experimental Infection of* M. tuberculosis*

**Figure 3:**
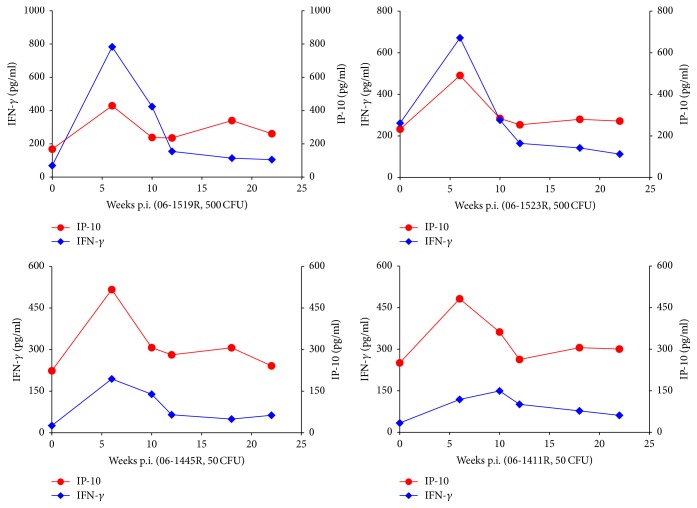
Dynamic changes of plasma IP-10 and IFN-*γ* in experimental tuberculosis infections.

For 4 monkeys with experimental infection of* M. tuberculosis*, plasma of 5 time points was used to detected the concentrations of IP-10 and IFN-*γ* (Figure 3). The dynamic changes of plasma IP-10 were consistent with IFN-*γ* in all 4 monkeys, showing a transient increase during the infection period. The peaks of IFN-*γ* in high dose treated monkeys (06-1519R and 06-1523R, 500 CFU) were significantly higher than low dose treated monkeys (06-1445R and 06-1411R, 50 CFU). However, differences of the peaks of IP-10 between monkeys were not significant.

### 3.4. Release of IP-10 and IFN-*γ* Is Induced by Incubation of Whole Blood from Monkeys with Naturally Acquired Tuberculosis with PPD

**Figure 4:**
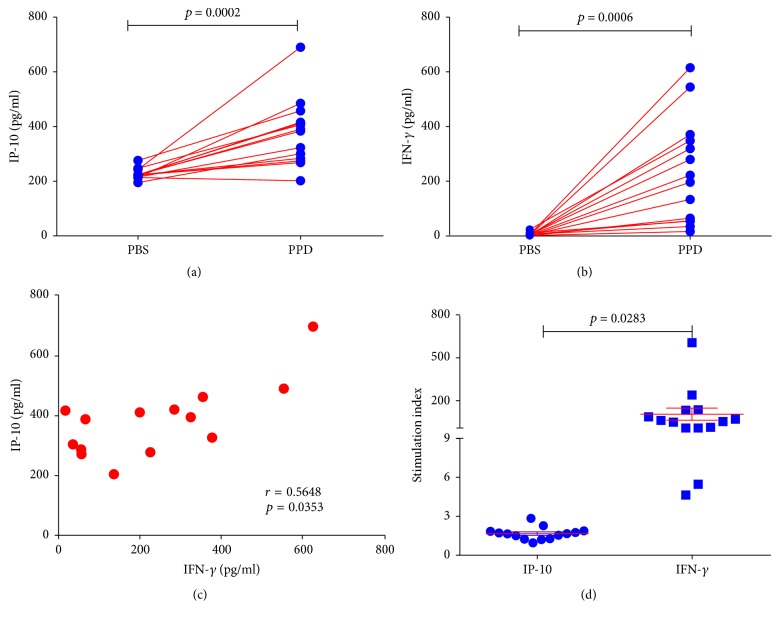
*Results of whole blood assay stimulated with PPD*. Increased IP-10 and IFN-*γ* plasma concentrations were observed after stimulation (a and b). (c) showed a high positive correlation between IP-10 and IFN-*γ* in plasma after incubation. Significant difference was found in SIs between IP-10 and IFN-*γ* in plasma after incubation (d).

Heparinized whole blood from 14 monkeys with naturally acquired tuberculosis was incubated with PPD and PBS. IP-10 and IFN-*γ* plasma concentrations were determined and compared to those of preincubation (Figures 4(a) and 4(b)). Significantly elevated IP-10 and IFN-*γ* levels were observed in plasma after incubation with PPD (*p* < 0.05). Spearman's rank correlation coefficient analysis showed a high positive correlation between IP-10 and IFN-*γ* in plasma after incubation (*r* = 0.5648, *p* = 0.0353) (Figure 4(c)). Furthermore, we evaluated the stimulation index (SI) of IP-10 and IFN-*γ*, which was calculated relative to preincubation. The SI of IFN-*γ* was significantly higher than IP-10 (*p* < 0.05) (Figure 4(d)). Results revealed that IFN-*γ* was more suitable as evaluation index for tuberculosis diagnosis by whole blood assay incubated with PPD (also named whole blood IFN-*γ* assay).

## 4. Discussion

As an important proinflammatory chemokine, IP-10 is not only involved in leucocyte migration and activation but regarded as a promising candidate as surrogate biomarker for diagnosis and therapy responses in tuberculosis patients [13]. IFN-*γ* is one of the most important proinflammatory cytokines in many infectious or noninfectious chronic inflammatory diseases, and whole blood IFN-*γ* assays are popular in diagnosis of human tuberculosis and bovine tuberculosis [6, 14–16]. However, few research efforts were focused on IP-10 in nonhuman primates. Only a small amount of studies employed whole blood IFN-*γ* assays to diagnose tuberculosis in nonhuman primates [8–11]. This study was set to investigate the correlations between IP-10 and IFN-*γ* (protein) responses in healthy and tuberculosis monkeys.

In this study, marked changes in both IP-10 and IFN-*γ* responses during infection with tuberculosis were observed in monkeys with naturally acquired tuberculosis compared with healthy controls. Dynamic changes of IP-10 and IFN-*γ* responses in monkeys with experimental infection of* M. tuberculosis *showed a significantly transient increase compared to the initial values. Positive correlations between IP-10 and IFN-*γ* were observed in healthy monkeys and monkeys with either naturally acquired or experimental infection of* M. tuberculosis*. After PPD stimulation, IP-10 release in whole blood of monkeys with naturally acquired tuberculosis was highly correlated with that of IFN-*γ*, showing significantly elevated plasma IP-10 levels. Though significantly increased plasma IP-10 levels were observed in monkeys with either naturally acquired or experimental infection of* M. tuberculosis* and PPD stimulated whole blood of monkeys with naturally acquired tuberculosis, the stimulation index (SI) was significantly lower than that of IFN-*γ*. Our findings based on a small amount of clinical samples do not support IP-10 as a plasma biomarker for immunodiagnostic assays of nonhuman primate tuberculosis by detecting the immune activation of T-lymphocytes in memory response to stimulation with tuberculin antigens (PPD). However, IFN-*γ* release in whole blood of monkeys is a sensitive marker of immunodiagnostic assays of tuberculosis.

Previous studies have identified IFN-*γ* release as a sensitive and promising diagnostic biomarker of* M. tuberculosis* infection in nonhuman primate [8–11], and our findings support the previous studies. However, data on IP-10 in this setting of nonhuman primates are more limited. IP-10 has been investigated in human tuberculosis patients and bovine tuberculosis. Most reports supported that the diagnostic accuracy of IP-10 was to be on par with IFN-*γ* release assays (IGRAs) [6, 17–20].

On the contrary, some studies on bovine tuberculosis revealed that IP-10 protein was not suitable as a biomarker for bovine tuberculosis using the current testing protocol and reagents [5]. Our finding showed increased plasma IP-10 levels after PPD stimulation of the whole blood from monkeys with naturally acquired tuberculosis, but the SI was lower than that of IFN-*γ*. The following should be taken into consideration: (1) the sample size of this study was small; (2) repeated tuberculin skin testing of monkeys for tuberculosis identification at two-week interval might affect the IP-10 release. Though there was no evidence that repeated tuberculin skin testing could affect the IP-10 release in monkeys with tuberculosis, some reports identified the effect of repeated tuberculin skin testing of cattle on immune responses and disease of bovine tuberculosis [21, 22].

For monitoring inflammation associated with tuberculosis infection of nonhuman primates, plasma IP-10 and IFN-*γ* can be used as biomarkers. Judging from the diagnostic performances of whole blood stimulation assays in this study, the IFN-*γ* release assay is more valuable than that of IP-10. However, whether IP-10 can be used as diagnostic biomarker of nonhuman primate tuberculosis still requires further investigation by more clinical samples.
